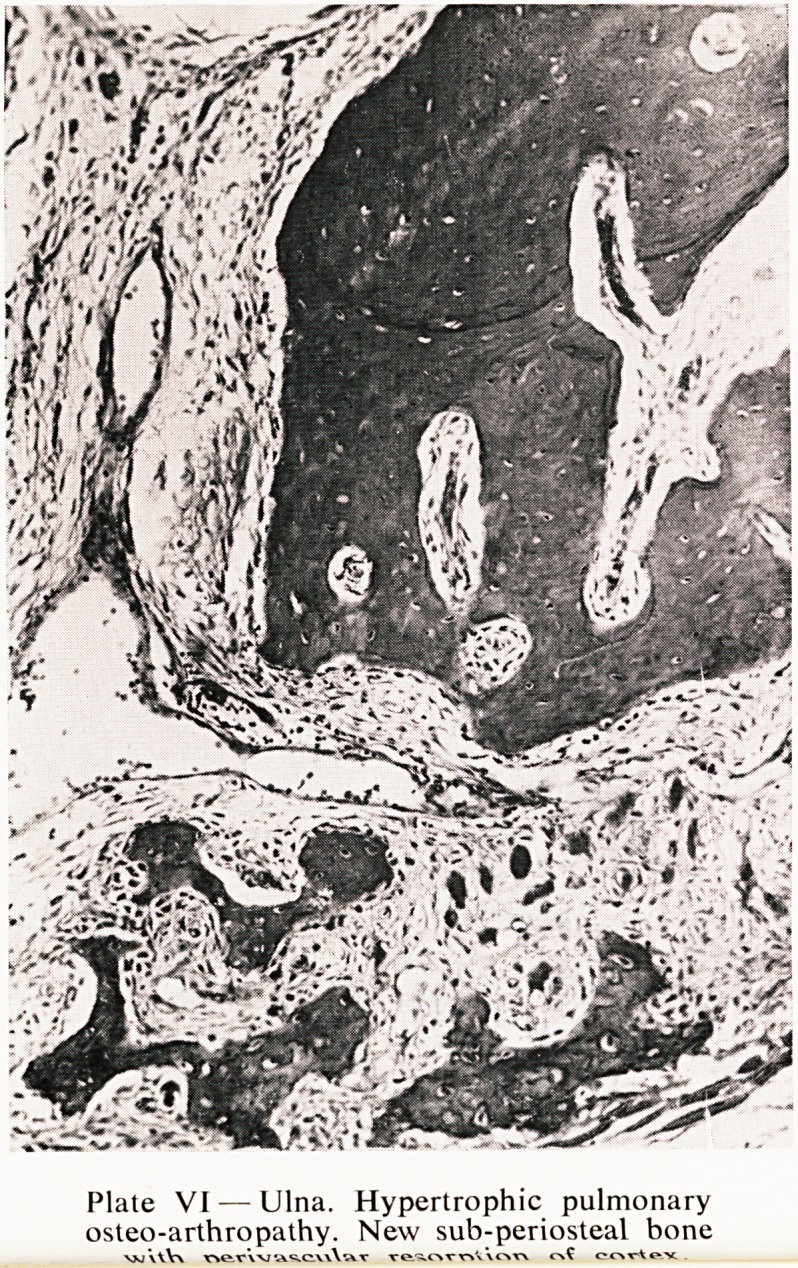# Carcinoma of the Lung with Cushing's Syndrome

**Published:** 1967-01

**Authors:** T. F. Hewer


### CARCINOMA OF THE LUNG WITH CUSHING'S SYNDROME

*A Clinico-Pathological Conference held in the University of Bristol on 19th October,* 1965

Chairman: Professor T. F. Hewer

P.M. No. 9083

*Dr. Read:* It is well recognised that some people have neoplasms which produce hormonal effects. In some of these the hormonal effects may play a considerable part in the patient's illness, for instance secretion of insulin by an insulinoma. The tumour itself may play a relatively small part.

This case is I think, however, an example of a patient who has a tumour which is producing an endocrine effect, that has little to do with his clinical picture. This effect is difficult to pick up and needs special tests in order to show it. It is in fact the case of a man aged 66 who was a retired Esso pump man. He was sent up to Outpatients and seen by Dr. McCarthy with a letter from his doctor which stated " This man has, I think, got moderate heart failure and I would like you to see him. I have been treating him with digitalis and diuretics. B.P. 150/90 ". On going into the story this did fit cardiac failure. He had been short of breath increasingly so for the past six months; he had a productive cough with purulent sputum (about 250 ml. a day) and he had also noticed swelling of the ankles. However there was one very important point about his swollen ankles and that is that the swelling was painful. This is of course unusual in cardiac failure, unless the swelling is gross and of rather sudden onset. There had also been a decline in his general health and he had lost a stone or so in weight.

His other history was relatively unimportant. He smoked 20 cigarettes a day. Interestingly enough his father died of a lung cancer. In Outpatients he was seen to be a thin, ill and frail-looking man and he had signs of consolidation and collapse of the left upper lobe. He also had gross finger clubbing, and his hands were hot and pulsatile suggesting an increase of blood flow through them. He was pyrexial, had tachycardia, and brisk tendon jerks. There were no signs of glands anywhere in the axillæ or neck, and rectal examination was normal. Dr. McCarthy made a confident diagnosis of carcinoma of the bronchus, in view of the gross pulmonary signs and the fact that he was a fairly heavy smoker, and that he had lost weight and strength. He was admitted soon afterwards and investigations were done. His Hb was 77% and he had a raised ESR, 46 mm./hr. I think the first important investigation carried out, the localizing one, was the chest X-ray.

*Dr. Gordon:* An X-ray of the patient's chest shows a fairly extensive opacity in the upper part of the left lung. A lateral view makes it clear that the opacity occupies the whole of the upper lobe and that the lower lobe is normally translucent. This presumably means that there is a consolidation of the left upper lobe, and it is not possible to differentiate any separate mass in the lung or hilum, which might be acting as a primary cause for this.

The X-rays of his ankles show a layer of periosteal new bone, particularly along the fibula. Similar changes are to be seen in the hands and forearms and can just be identified on the radius and ulna. This appearance indicates hypertrophic pulmonary osteoarthropathy, which we know occurs occasionally in association with various diseases of the heart and lung, including neoplasm of the lungs.

*Dr. Read:* The diagnosis seemed fairly certainly one of carcinoma of the lung, and it looked as though the pain and swelling of the ankles was not simple œdema but was due to the bony changes.

But it doesn't do, these days, to stop there and accept the diagnosis of carcinoma of the lung. One wonders whether one is going to meet a hormone-producing tumour. So a few further tests were done, and it was shown that he had abnormal serum electrolytes. His serum potassium on one occasion was 2.7 and his plasma bicarbonate 34. His plasma chlorides were low, in other words he had a hypokalæmic alkalosis, the sort of thing that one gets with chronic potassium deficiency. The question was, why should he have this? If you go back in his history you will remember his doctor treated him with diuretics, and it is possible that his potassium deficiency was due to that. But we thought that we were dealing with something a little strange here, perhaps with a tumour that could produce potassium deficiency. So more tests were done. His liver function tests showed a raised serum alkaline phosphatase. It was uncertain whether this was due to liver secondaries, or bone involvement, or indeed both. His sputum contained numerous mixed organisms, but nothing very striking, despite the fact that it was fairly copious and purulent. His blood sugar was not done, although in retrospect it should have been, but certainly there was no glycosuria. His cardiogram was normal apart from partial right bundle-branch block. Bronchoscopy confirmed the findings about which you have already heard, and showed fixation of both major bronchi and considerable distortion of the left main bronchus; changes fully compatible with those of a bronchial carcinoma of the left upper lobe bronchus.

But the interest in this case, apart from the potassium findings, is in the plasma corticosteroid levels and the corticosteroid metabolism in general. He was an ill man. He was short of breath and had painfully swollen legs and therefore it was not possible to do a great deal in the way of investigation, but his 11-hydroxy-cortico-steroids in the plasma were measured by Dr. Keane on several occasions during the day and the levels obtained did not vary very much, being 20, 23 and 29 micrograms /100 ml., the normal being 6 to 24 micrograms/100 ml. So the patient's level was at the upper limit of normal, but I do not think you can say it definitely is abnormal. He had lost his circadian rhythm and that is abnormal; it is an early sign of adrenal hyperfunction.

The other thing that was done was of course to measure the 17-hydroxy-cortico-steroids in his urine. This again is largely representative of break-down products of cortisol. The normal level is 6 to 24 micrograms/24 hours, but this patient was passing 52 micrograms/24 hours, and so this level was considerably increased. It did suggest, therefore, that his adrenals were working overtime for some reason.

We next tried to find out whether we could suppress this raised level of 17-hydroxycortico-steroids in the urine, with dexamethasone. If you suspect that a patient has Cushing's syndrome with adrenal hyperplasia, it is usually possible with a sufficiently large dose of dexamethasone to produce some degree of suppression. If, however, the Cushing's syndrome is due to a tumour, or is due to the production of an ACTH-like substance from somewhere outside the pituitary, then suppression does not occur. For 48 hours he was given 2 mgm of dexamethasone 6 hourly, and the excretion fell from 52 to 31.7 micrograms in 24 hours. So there was some suppression. I think it would be best if we left that for the moment and discussed it later on, because it was unexpected.

Plate 1.—Right lung. Focal centrilobular emphysema.

Plate. II.—Right lung. Enlargement shows detailed pattern of emphysema.

Plate III.—Left lung. Carcinoma of upper lobe, compression collapse of lower.

Plate IV.—Lung tumour. Adenocarcinoma.

Plate V — Metastasis. Well differentiated mucus-secreting tubules.

Plate VI — Ulna. Hypertrophic pulmonary osteo-arthropathy. New sub-periosteal bone with perivascular resorption of cortex.

There therefore seemed some evidence in this patient that the adrenal glands were hypertrophic, and in a patient with a carcinoma of the lung it is not uncommon to find this. There are perhaps 30 or so cases of carcinoma of the lung on record with adreno-cortical hyper-activity, and other types of tumour outside the lung (œsophagus, stomach, pancreas, etc.) will do the same sort of thing.

Although we were very interested in the high steroid levels, these made little difference to the patient. We referred him for some X-ray therapy, and though he had a few treatments of this he began to go rapidly downhill. One thing we did accomplish that is of interest in retrospect; we did correct his serum potassium, and this is usually difficult to do in someone with an endocrine cause for potassium depletion. Anyway he died, we thought, of carcinoma of the lung, probably of the oat-cell type. It had produced consolidation of the left upper lobe; it had probably produced bony, and possibly hepatic metastases (because of the abnormal liver function tests); it was producing adrenal hyperplasia and therefore he was suffering also from a secondary type of Cushing's syndrome. Perhaps at the end he got an overwhelming pulmonary infection. This would not be surprising in someone with bronchial obstruction, on X-ray therapy, and in poor general health.

*Dr. Knapp:* I notice the serum sodium was 128 mEq/litre, which is well below the accepted normal. We have in the past seen patients who have produced excessive amounts of anti-diuretic hormone or a substance like anti-diuretic hormone apparently from a tumour, normally of the lung. They had sodiums of this level and lower. I wonder if you would consider this a possible explanation for this patient's low serum sodium, and the rather low electrolytes and plasma proteins that he had?

*Dr. Read:* I would agree that he had a low serum sodium, but I thought that when he came in that he had a normal one. I commented on that, you see there is one of 134 mEq/litre and I think the one you are talking about was a terminal one. It is very difficult to make statements about the serum sodium levels in someone who is moribund, because the level often goes down. I think it is some evidence of impending dissolution. Would you agree?

*Dr. Knapp:* I quite agree with the last remark.

*Dr. Froud:* I wonder if I could ask Dr. Read in what order these electrolyte changes occur?

*Dr. Read:* Are you talking about people who have low serum sodium or people who have excess ACTH production?

*Dr. Froud:* The latter.

*Dr. Read:* Well I presume the only changes there should be are those related to potassium metabolism. I wouldn't have expected any change in serum sodium as a result of excess cortisol production. I do not think it would be increased. There might be more sodium on board and the patient would probably become a little œdematous. I think the changes are merely those of potassium deficiency but of course they are relatively uncommon in ordinary Cushing's syndrome. This is not ordinary Cushing's syndrome, this is very extraordinary Cushing's syndrome, and these patients tend to have adrenals working even harder than those in ordinary Cushing's syndrome, and they are the ones who often become hypokalæmic.

*Dr. Pearson:* Interesting, if their suprarenals are almost destroyed by the secondaries.

*Dr. Read:* Yes, because this chain of events does not depend on secondaries, but I suppose you could have secondaries and hyperfunction together. It would be interesting to think that perhaps, in time, if the patient lived long enough, the secondaries would destroy the hyper-functioning adrenal.

*Dr. Pearson:* I am wondering whether we have missed some of these cases because the adrenals have been destroyed.

*Dr. B. Hale:* I should like to ask Dr. Read what he thinks is the usual outcome of these cases, in patients who live long enough? Am I right in thinking that if one could remove the malignancy surgically, or deal with it adequately by radiotherapy, then the process is quite often reversible?

*Dr. Read:* That is very difficult to answer. I think one of the things that would happen, if the patient lived long enough is that he would get the body changes of Cushing's syndrome. I think these are not there because the patient does not survive long enough. People have had tumours removed and the cortisol levels have fallen. People have had tumours irradiated and the cortisol levels have fallen, with some amelioration of the clinical picture. There are certain parts of the picture which are clinically important. The one which is particularly important, I think, is the onset of psychosis. If the patient is mad then this is treatable with radiation or surgery of the primary tumour. Of course the other thing you can do is to treat these patients with metapyrone and turn off the cortisol ' tap '. That again is therapeutically successful in a patient who has something like a psychosis, or perhaps gross uncontrollable diabetes. I do not know what happens if they live a long time. I do not think they usually do.

*Professor Hewer:* I think it is time Dr. Lloyd gave us his findings.

*Dr. Lloyd:* I don't think I should have recognised this subject as suffering from Cushing's syndrome. He was emaciated, he had a brownish livid hue, and there was some scaling of the skin around the neck and the upper part of the chest. There was also severe clubbing of the fingers and toes, as you have already been told. I didn't like to take one of his fingers for section, but I took one of his toes instead and you can see it underneath one of the microscopes. The changes supposed to be seen there, characteristic of this hypertrophic pulmonary osteoarthropathy, are an increased vascularity and increase of the connective tissues which tends to be rather œdematous. I think it probably does show these changes, but I did not find them very convincing, possibly because I did not have a control to look at at the same time.

The peritoneal cavity was normal and so was the right pleural cavity, but the left contained 400 ml. of fluid which smelt of formalin. On inspecting the other viscera it was apparent that formalin had been injected into the lung *post mortem,* resulting in partial fixation of the lungs, stomach and upper loop of jejunum.

The heart was normal. The mouth and pharynx were beautifully fixed; the larynx, trachea and bronchi contained mucopus which had also been fixed, so that bacteriological examination was unprofitable.

The lungs showed some very interesting changes. On the right side there was a considerable degree of focal centrilobular emphysema (Plates I and II). The peribronchial fibrosis tends to block up the lymphatic channels, producing retention of carbon pigment, more particularly in the places where there is emphysema rather than in the intervening places which tend to remain pink.

Plate III shows on the left the carcinoma that has been described to you from radiological appearances. There is a good deal of compression collapse of the lower lobe because of the effusion that I have described, and the other lobe is almost entirely replaced by malignant tumour which showed a very considerable degree of necrosis.

What was rather surprising (Plate IV) when we came to look at the sections was to find that this was not an oat cell carcinoma. It was not an anaplastic carcinoma, it was not a squamous carcinoma, but it was in fact adeno-carcinoma. There was so little of it actually surviving in the lung that it was necessary to examine the metastases in order to appreciate the full beauty of the tubular differentiation (Plate V).

The adrenal glands were indeed very large; the right adrenal weighed 15 g. and the left adrenal 10 g. the normal being considerably less than half of that. And the reason why they were enlarged was principally because they were filled with secondary carcinoma. But Dr. Read nevertheless was perfectly right; he had foretold that there would be hypertrophy of the cortex, and this was present. On the surface the glomerular zone was hardly increased in size at all but the zona fasciculata was very much increased and the zona reticularis was just a little increased as well. That, I believe, is characteristic of this condition, because only those parts of the adrenal cortex enlarge that are stimulated by the hormonal substance which is being produced by the primary tumour.

The lymph nodes contained metastases all the way from just below the bifurcation of the trachea up to the thyroid. Also there was metastatic carcinoma in the spine. But the liver showed very small and not very numerous metastases, mainly on the surface.

Dr. Read pointed out to you, that when you get a raised alkaline phosphatase it may be because there is too much being produced or it may because it is not being destroyed by the liver. In the present case there was plenty of liver to have destroyed any normal quantities of alkaline phosphatase and therefore there must have been excessive production. You can get excessive production in the presence of secondary tumour, provided there is a fair degree of reaction of bone going on at the same time, so that there is new bone being formed. I do not know whether there was a tremendous amount of that in this particular instance but of course we do have evidence from the X-rays that there was a lot of excess bone formation going on in connection with the hypertrophic pulmonary osteoarthropathy. You see increased bone formation underneath the periosteal surface of some of the bones.

Dr. Lloyd then showed a transverse section of the ulna, which was the bone Dr. Gordon demonstrated in his X-rays and therefore the one that was taken for microscopical section; the normal part of the bone is only to be seen at one edge where the periosteum has been stripped. From there onwards it becomes abnormal and there is rather finer structure and more purplish-coloured bone which has been laid down on the external surface of the cortex along the outer side. Moreover the cortex itself is very much more porous than it should be in consequence of the enlargement of the Haversian canals which are very much more prominent than usual and contain some fibrous tissue as well. A higher power shows details of the new periosteal bone and porotic cortex (Plate VI).

The testes showed atrophy. There was increased thickening of the basement membrane and differentiation does not get anywhere past the spermatocytes.

The pituitary gland was rather surprisingly normal, and showed no evidence of primary excess ACTH production. The acidophil cells were normal in their distribution, just a bit small, perhaps, but of the ordinary density. Of the granulated PAS-positive cells, the finely granular ones (basophils) were normal. The sparsely granulated rather coarsely grained mucoid cells (which are a variety of chromophobe) were more numerous than usual, but were not as big as they are in some cases of myxœdema; I do not really know what that means in this case. "S" granules were looked for. These are the granules which are believed to carry ACTH and these were totally absent, which shows that there was maximal suppression of ACTH production in the pituitary. This was caused by the excess of cortisone arising from the enlarged adrenal glands. The pineal gland appeared normal, but there may have been a little thickening of some of the glial fibrils.

So much for the findings. Now what does it all mean? As far as I am concerned the finding of the well differentiated adenocarcinoma was rather a surprise, particularly as it isn't generally regarded as being one of the growths connected with smoking. It was quite definitely one of the mucin-producing tumours. Of the 18 patients reported in the literature who had carcinoma of the lung associated with excessive ACTH production, 12 had oat-cell growth, 2 had anaplastic growths, 2 had poorly differentiated adenocarcinomas; Dr. Read's two cases (which he has already published) were of oat-cell carcinoma. The present patient seems to provide the only instance of such a tumour with good tubular differentiation and mucin production. I think he probably did have some chronic bronchitis in spite of the fact that the usual changes (increase of goblet cells in the smaller broncioles) were not present, for though there was not much fibrosis around the terminal bronchioles, there was some.

*Dr. Knapp*: I wonder if I could ask Dr. Read if he thought the man was pigmented as Dr. Lloyd described his skin as brownish and livid. This would be very interesting, because we know that the melanophore stimulating hormone consists of the first 27 or so aminoacids that make up ACTH, if this patient was producing excess ACTH perhaps he was producing excessive melanin-stimulating hormone, too.

*Dr. Read:* It is a nice story. I cannot say yea or nay. We did not think he was very pigmented. He had had radiotherapy, and that might have increased the pigmentation on his chest but it should not have caused generalised pigmentation. No comment was made in the notes that he had more pigmentation than any other 65 year old man might have who had spent most of his time on a petrol pump outdoors.

*Dr. Lloyd:* I didn't feel I could comment on it. I was merely noting it as fact, I'm afraid. I don't think it was melanin.

*Dr. Read:* Perhaps somebody would like to comment on why we were able to suppress this man's 17 hydroxy-cortico-steroids by 20 to 30 mgms in 24 hours with dexamethasone?

*Dr. Keane* You did get them down from 52 to 32. I think that if one had followed this man's urinary steroids for several days one might have seen this variation anyway. This sort of variation from 50 to 30 may not mean anything. The point I would like to make is that if anybody gets one of these cases and manages to treat it successfully by surgery the patient should be provided with some steroid cover, because the patient might well go into an acute Addisonian condition if the ACTH producing tumour is removed.

*Professor Hewer:* There was not much chance of this tumour being removed.

*Dr. Keane:* No. But possibly somebody is going to find an operable case or a treatable case. It is a distinct possibility.

## Figures and Tables

**Plate I. f1:**
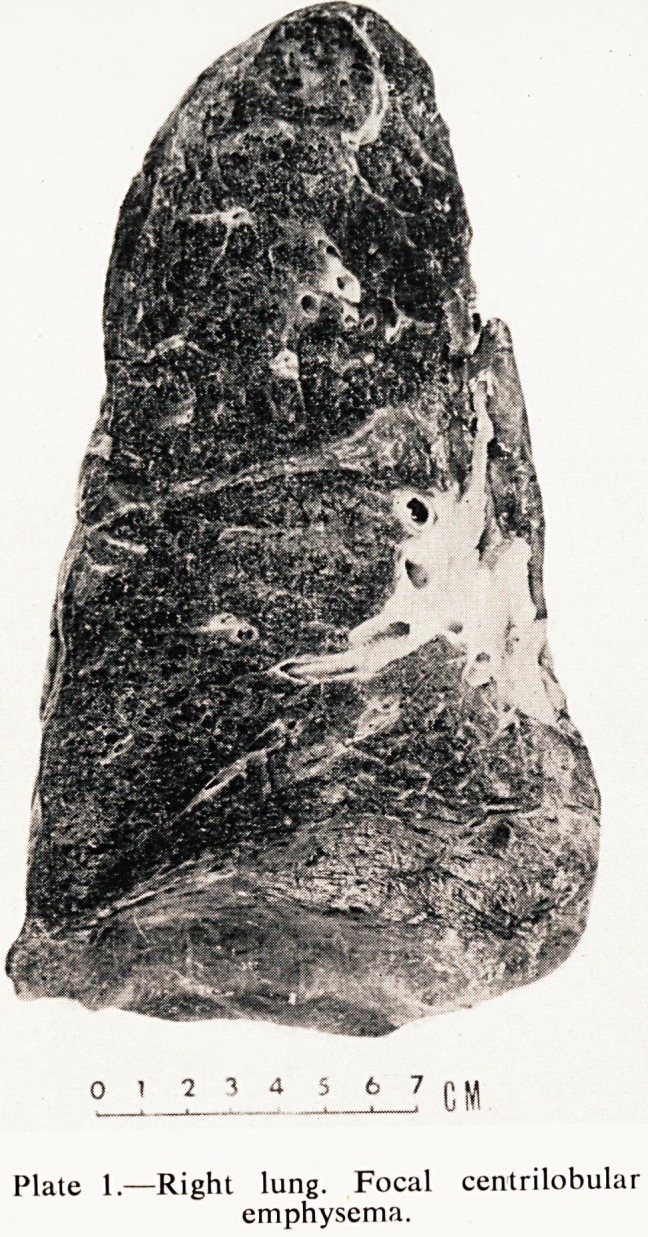


**Plate. II. f2:**
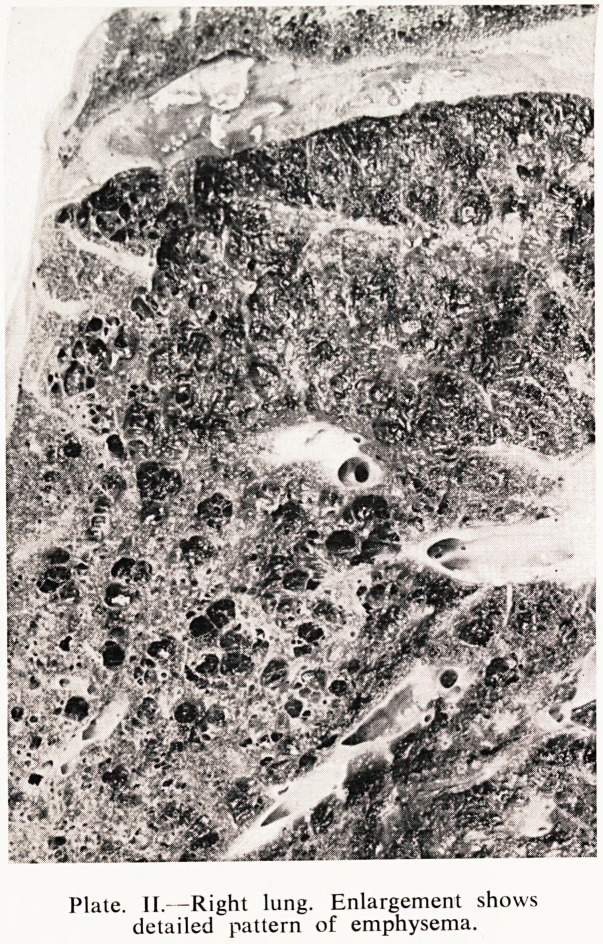


**Plate III. f3:**
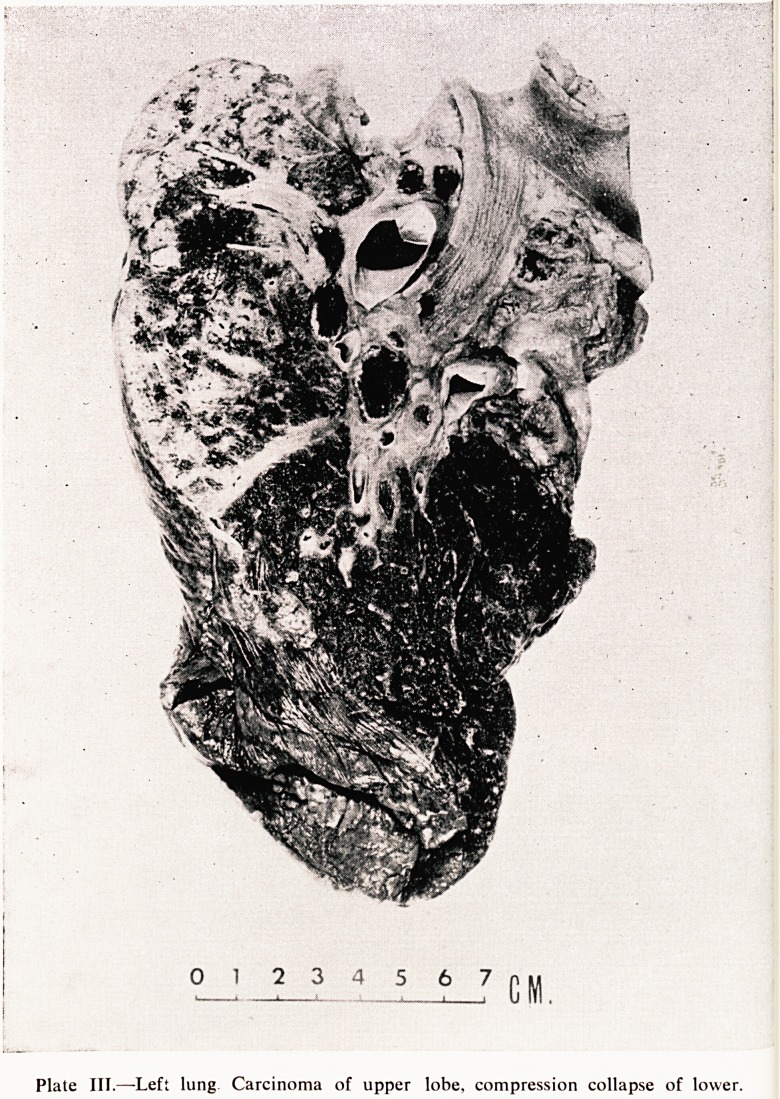


**Plate IV. f4:**
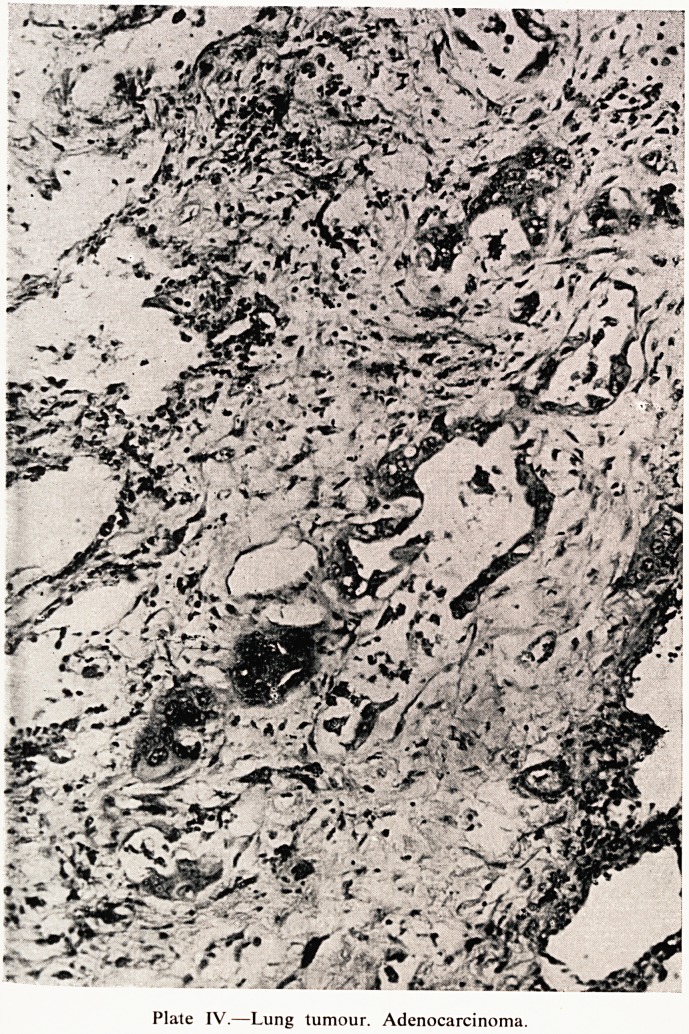


**Plate V f5:**
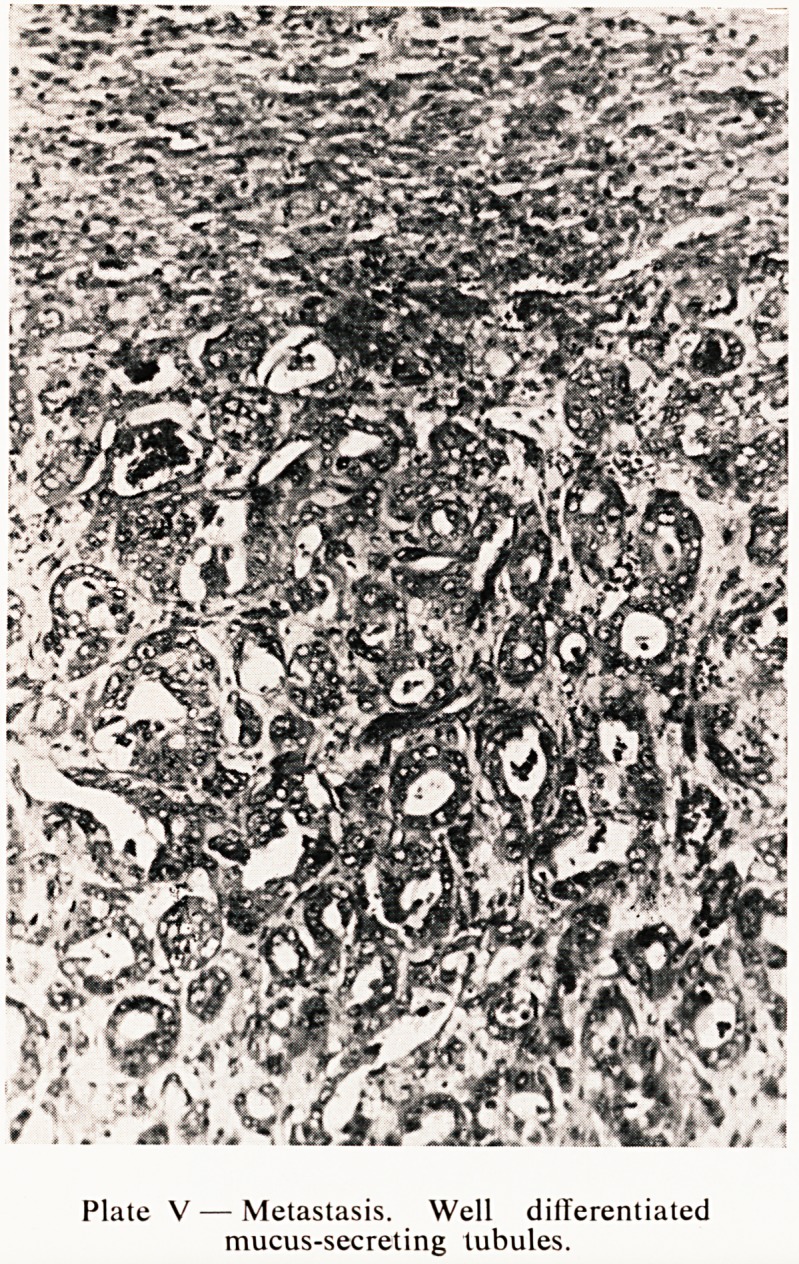


**Plate VI f6:**